# Surgical and audiological outcomes with a new transcutaneous bone conduction device with reduced transducer thickness in children

**DOI:** 10.1007/s00405-023-07927-9

**Published:** 2023-03-31

**Authors:** Kerstin Willenborg, Thomas Lenarz, Susan Busch

**Affiliations:** 1 Department of Otolaryngology, Hannover Medical School, Carl-Neuberg-Str. 1, 30625 Hannover, Germany; 2 Cluster of Excellence H4A, Hannover, Germany

**Keywords:** Bone conduction implant, Children, Conductive hearing loss, Bone thickness, BCI 602

## Abstract

**Purpose:**

Due to smaller bone thickness, young children with conductive or mixed hearing loss or single-sided deafness were previously most commonly treated with a percutaneous osseointegrated bone-anchored hearing aid (BAHA) or an active middle-ear implant. While the BAHA increases the risk of implant infections, skin infection, overgrowth of the screw or involvement of the implant in head trauma, middle-ear implant surgery involves manipulation of the ossicles with possible risk of surgical trauma. These complications can be omitted with transcutaneous bone conduction implant systems like the MED-EL Bonebridge system. The purpose of this study was to analyze whether the second generation of the Bonebridge (BCI 602) that features a decreased implant thickness with a reduced surgical drilling depth can be implanted safely in young children with good postoperative hearing performance.

**Methods:**

In this study, 14 patients under 12 years were implanted with the second generation of the Bonebridge. Preoperative workup comprised a CT scan, an MRI scan, pure tone audiometry, or alternatively a BERA (bone conduction, air conduction). Since children under 12 years often have a lower bone thickness, the CT was performed to determine the suitability of the temporal bone for optimal implant placement using the Otoplan software.

**Results:**

All patients (including three under the age of five) were successfully implanted and showed a good postoperative hearing performance.

**Conclusion:**

With adequate preoperative workup, this device can be safely implanted in children and even children under 5 years of age and allows for an extension of indication criteria toward younger children.

**Supplementary Information:**

The online version contains supplementary material available at 10.1007/s00405-023-07927-9.

## Introduction

Within the first 5 years of life, stimulation of the auditory pathway is essential to take advantage of the high level of auditory plasticity for speech and language development. In cases of hearing impaired infants and small children, early intervention is, thus, crucial to language development, social skills, and success in school. Surgical hearing restoration or the use of conventional hearing aids is often limited or impossible [1–4], especially in children with severe conductive or mixed hearing loss or single-sided deafness often due to malformations of the outer or middle ear including microtia, isolated atresia of the external ear canal or acquired defects, such as chronic otitis media and cholesteatoma. In these children, auditory intervention usually starts with bone conductive devices fixed to the head by a headband [5] or by an adhesive adapter (Adhear MED-EL, Innsbruck, Austria) [6]. A major drawback of this non-surgical therapy is that skin and soft tissue between transducer and bone limit the amount of energy that can be effectively transmitted, thus leading to low hearing levels and/or poor sound quality. Furthermore, in case of the headband option, constant pressure must be applied to the skull to provide good transmission, causing discomfort and in some cases rejection of the device by the child [7–9]. Surgical options in children with a smaller bone thickness previously mainly comprised a percutaneous osseointegrated bone-anchored hearing aid (BAHA) due to its low drilling depth into the bone, or an active middle-ear implant with a low profile [10, 11]. A middle-ear implant cannot always be applied in children with conductive hearing loss due to anatomical reasons [12]. Furthermore, middle-ear implant surgery involves manipulation of the ossicles or inner ear with possible risk of surgical trauma and post-operative implant displacement especially in children with more severe malformations, where surgical treatment can be more challenging and the risk of injury to important anatomical structures is higher [12–14]. In percutaneous hearing systems like the BAHA connect (Cochlear, Sydney, Australia) or the Ponto (Oticon Medical, Askim, Sweden), a titanium implant is anchored in the mastoid bone and attached to a skin-penetrating abutment that is coupled to a vibration transducer [11]. In Europe, the minimum age for this type of device is not regulated by law, although based on clinical experience many centers recommend application only beyond 5 years of age or with a minimum bone thickness of 3 mm [15]. Although effectiveness in terms of hearing benefits is high in bone-anchored hearing aids, they feature several limitations. The percutaneous character of these systems facilitates the involvement of the implant in head trauma and necessitates the prevention of skin overgrowth and comprehensive daily care with potentially high infection rates especially in children [16, 17]. Further complications are implant displacement or loss of the implant due to insufficient osseointegration, possibly leading to revision surgery or even explantation [16, 17]. The revision surgery rate due to fixture loss is higher in children with smaller bone thickness [15]. This holds true especially in children with genetic defects where the bone of the skull may develop slowly. In transcutaneous bone conduction implants like the Bonebridge (MED-EL, Innsbruck, Austria), the first implant generation (BCI 601) was approved for implantation in children of age 5 and older in 2014 (Online Resource 1). The implant body including the stimulator is placed in the bone under the skin. The system’s processor is held in place magnetically on the intact skin over the implant and wirelessly transmits the stimulation signals to the implant. This system composition hence avoids possible infections and the necessity to prevent skin overgrowth and also overcomes many other disadvantages of percutaneous systems [13, 18–21]. Furthermore, no osseointegration is necessary facilitating earlier activation [13]. However, the implant has to be inserted into the bone, which requires drilling of a bone bed. Thus, sufficient bone thickness remains an issue. Thin skin and low thickness of the mastoid bone, which lead to protrusion of the implant, often prevented implantation of the BCI 601 in younger children. In contrast thereto, downsizing of the transducer and design optimization of the second generation Bonebridge (BCI 602) allows for a complete fit of the implant body into or behind the mastoid bone without compromising the dura and sigmoid sinus, thus facilitating implantation in young children as well (Online Resource 1).

The objective of this study was the evaluation of the preoperative imaging, surgical procedure and audiological results of the second generation of the Bonebridge in children under the age of 12.

## Materials and methods

### Study subjects

The study comprises a retrospective analysis of 14 children (8 female, 6 male) implanted with the Bonebridge BCI 602 at the Hannover Medical School, Hannover, Germany between Oct 2019 and Sept 2021. All children suffered from a conductive hearing loss due to ear canal atresia or stenosis, middle ear dysplasia, or malformation. The Bonebridge was chosen for implantation after thorough clinical examination including imaging and audiological measurements and after considering the alternatives mentioned in the introduction. The mean age was 6.6 years (range from 3.2 to 11.6 years) with three children under the age of five at the time of implantation. Four children were simultaneous bilaterally implanted, and the right ear was randomly selected to be included in the analysis of audiological data. An overview of all children included in this retrospective study is presented in Table 1.

**Table 1:** Demoscopy of children

Patient ID	Sex	Implanted ear	Age at implantation (years)	Min. Bone thickness (mm)	BC_pre_ PTA4 (dB nHL)	LL_pre_ PTA4 (dB nHL)	Etiology
P01	Female	R	3.2	4.2	20^B^	90^B^	Ear canal atresia Middle ear dysplasia
P02	Male	R	4.4	7.5	5	63.8	Ear canal atresia
P03	Male	L	4.5	4.4	20^B^	60^B^	Ear canal atresie
P04	Male	L	5.3	5.2	30^B^	50^B^	Ear canal atresia
P05	Male	R	5.4	6.0	12.5	DNT	Ear canal atresia
P06	Female	R	5.4	6.8	− 1.3	57.5	Ear canal stenosis
P07	Male	R	6.8	3.2	2.5	57.5	Ear canal atresia, Middle ear malformation
P08	Female	L	6.9	3.2	0	50	Ear canal stenosis
P09	Female	R	7.1	6.8	− 3.8	56.3	Middle ear dysplasia
P10	Female	R	7.8	6.0	16.3	50	Ear canal atresia (stenosis after reconstruction)
P11	Male	L	7.9	5.3	3.8	67.5	Ear canal atresia
P12	Female	R	8.0	6.4	8.8	61.3	Ear canal atresia
P13	Female	R	8.2	5.3	1.3	71.3	Ear canal atresia
P14	Female	L	11.6	4.5	10	76.3	Ear canal atresia

*B* BERA measurement, *PTA4* four-frequency pure tone average across 0.5, 1, 2, and 4 kHz

### The Bonebridge system

The Bonebridge is a transcutaneous bone conduction implant system that consists of the audio processor worn on the intact skin and an implanted part, the bone conduction implant (BCI). The BCI consists of a receiver coil, a demodulator and the bone conduction floating mass transducer (BC-FMT) that is implanted in the bone and fixed with screws through the anchor holes of the fixation wings of the BC-FMT. The downsizing and optimization of the design of the second generation of the Bonebridge decreased the necessary drilling depth of the bone bed from 8.7 mm (first generation, BCI 601) to 4.5 mm (second generation, BCI 602). If the drilling depth cannot be safely achieved or in case of an uneven skull, ‘lifts’ (1-mm spacers) can be placed under the fixation wings of the BC-FMT. Lifts allow for contact of the fixation wings with the skull surface while reducing the required drilling depth by increasing the portion of the implant protruding above the surface of the bone.

### Preoperative imaging and determination of the potential implant site

**Fig. 1:**
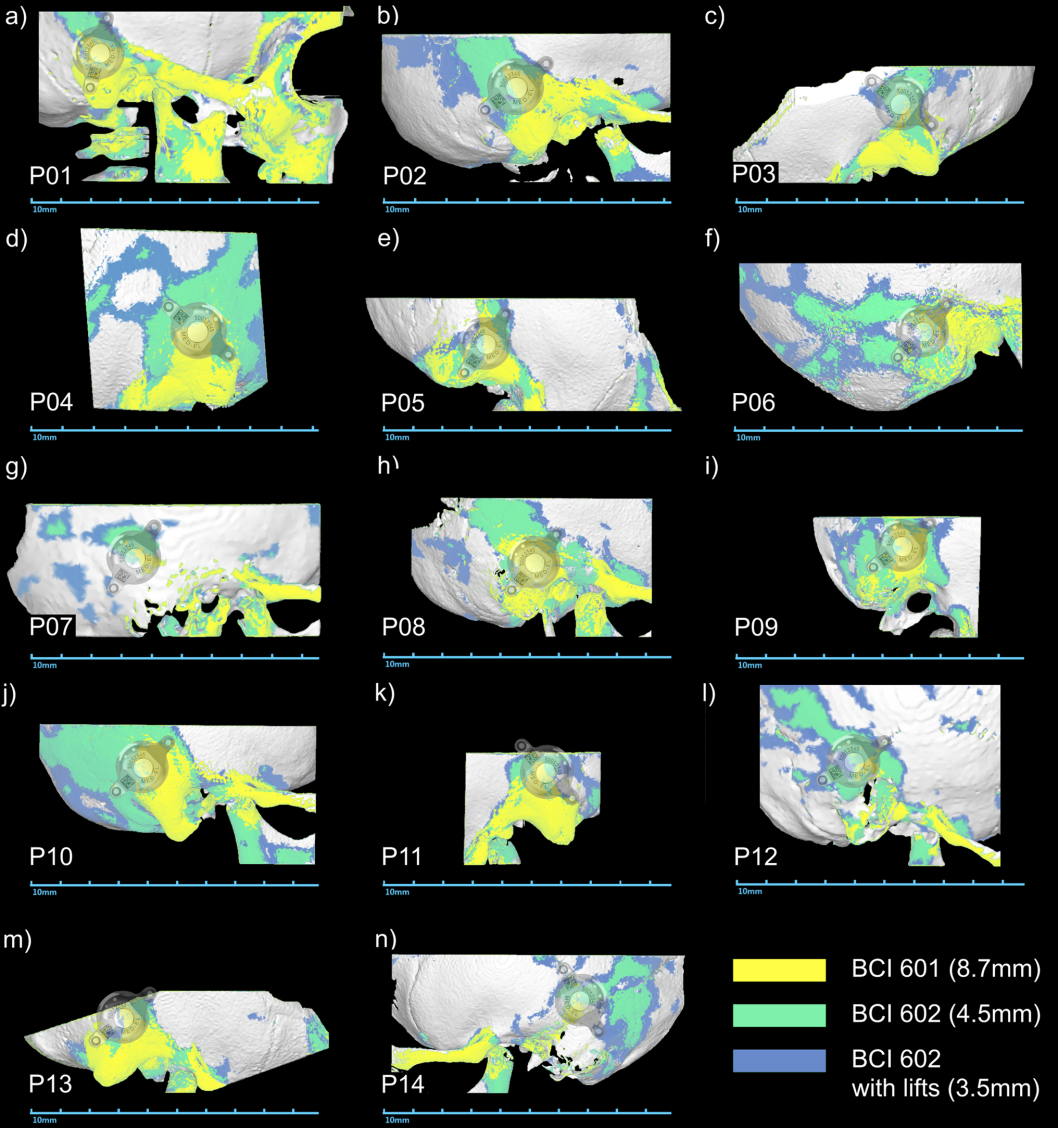
Bone thickness maps of all 14 children organized by age (a-youngest to n-oldest). The highlighted regions indicate where the bone is at least as thick as the bone bed that needs to be drilled for the BC FMT (the corresponding thickness values are stated in the figure legend). A projection of the BC-FMT indicates the optimal implant position in each panel

**Fig. 2:**
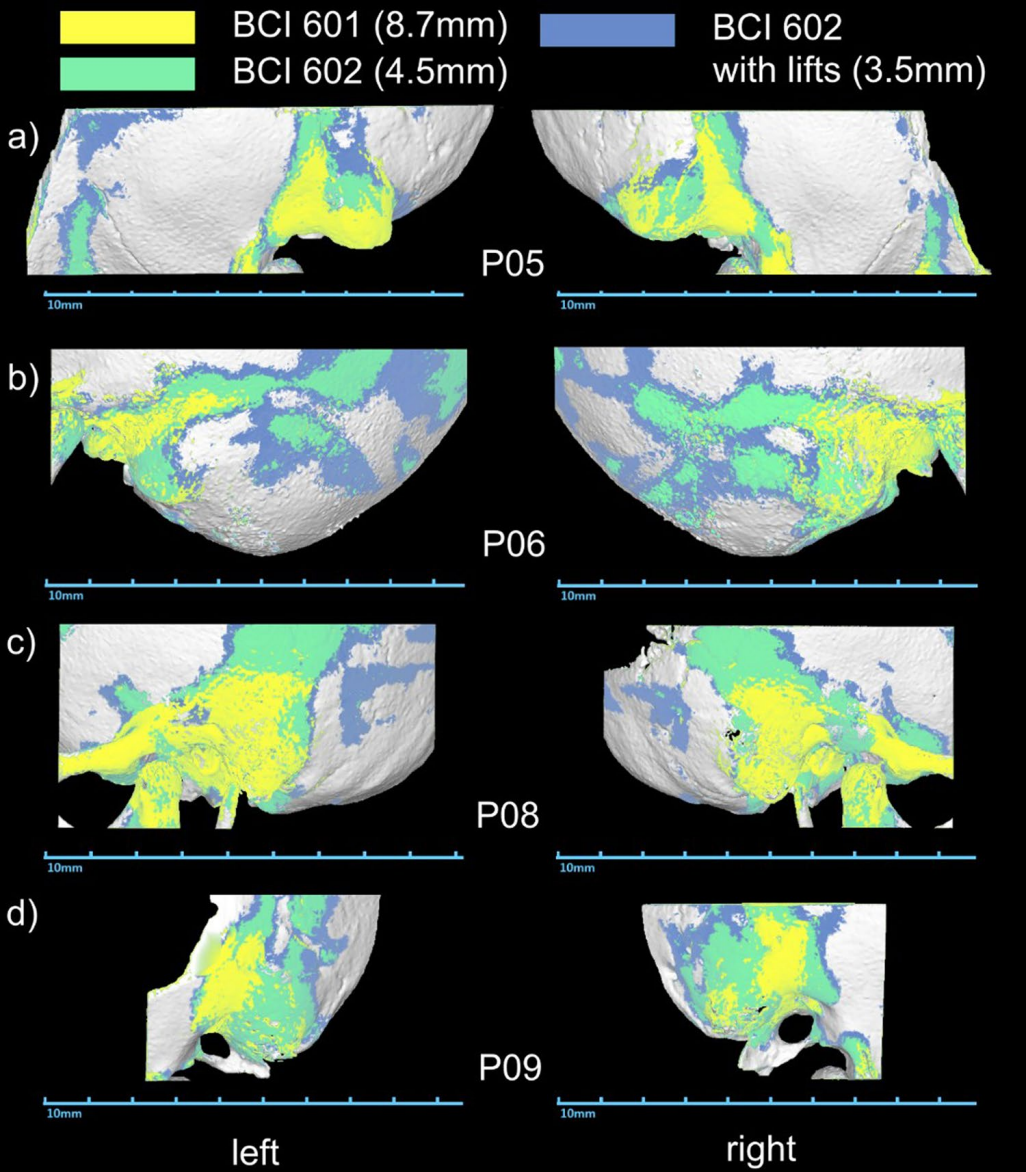
Bone thickness maps of the 4 children who were implanted bilaterally, organized by age (a-youngest to d-oldest). The highlighted regions indicate where the bone is at least as thick as the bone bed that needs to be drilled for the BC FMT (the corresponding thickness values are stated in the figure legend)

Before considering implantation, the absence of retrocochlear or central auditory disorders was confirmed by magnetic resonance imaging (MRI). Computed tomography (CT) scans with a standard temporal bone protocol are taken prior to every bone conduction implant surgery within the clinical routine at the Hannover Medical School. These scans are essential to determine if there are any anatomical contraindications and to define the optimal implant location for each individual patient. The latter includes the measurement of several bone thickness values of the temporal bone including the minimal bone thickness within the target area for implantation and under consideration of the transducer diameter and screw distance. The smallest value for each patient was noted for comparisons within the present study. Furthermore, the individual CT scans were retrospectively loaded into the otologic planning software Otoplan (Cascination AG, Bern, Switzerland) and investigated using the *Temporal Bone* module (Figs. 1, 2). This investigation consists of an automatic segmentation of the temporal bone and the subsequent computation of the temporal bone thickness. The temporal bone regions whose thickness values lie above a certain threshold can be highlighted in the software, and the corresponding images containing these thickness maps can be exported. Within this study, we created these images for each one of the 18 implanted ears for the thresholds of 8.7 mm, 4.5 mm, and 3.5 mm. These thresholds correspond to the bone bed depths necessary for the first generation of the Bonebridge (BCI 601), the second generation (BCI 602) and the second generation with 1-mm lifts, respectively. In order to allow for a direct comparison of different thickness maps of the same ear, the corresponding images were saved and imported into the open-source vector graphics editor Inkscape (version 1.1.0, Inkscape Project, available at https://inkscape.org) where different maps could be extracted, re-colored, and superimposed.

### Surgical procedure

**Fig. 3:**
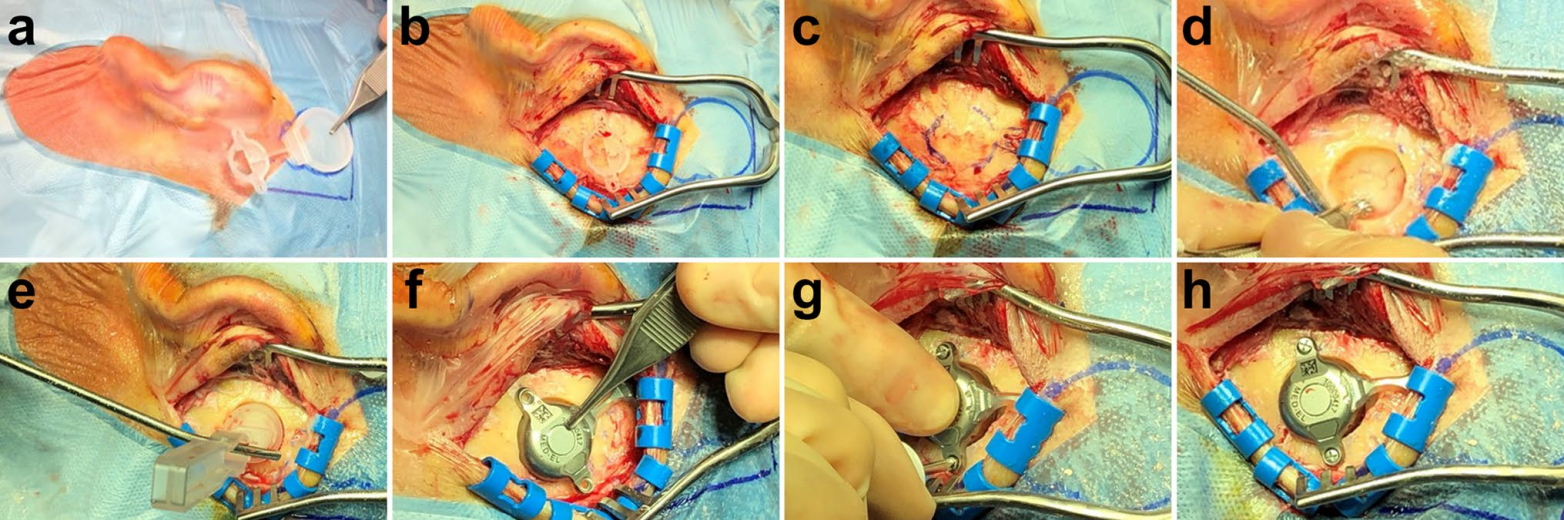
Surgical procedure. **a** Marking of the position of the BC-FMT and coil on the skin. **b** Checking of the position of the BC-FMT with the FP-sizer. **c** Marking of the position of the BC-FMT on the skull. **d** Drilling of a bonebed (4.5 mm deep). **e** Checking of the depth of the bonebed with the T-sizer. **f** The fixation wings should have flat contact with the bone. **g** Fixation of the implant with self-cutting screws. **h** Fixed implant in correct position before three-layered closure

Surgery was performed under general anesthesia following the guidelines of the manufacturer. Briefly, prior to operation, computed tomography (CT) scans were performed for all patients to detect aberrant anatomical structures like an atypical sigmoid sinus which were omitted as potential implantation sites and to measure bone thickness. Furthermore, Otoplan was employed to determine the regions where the implant could be positioned, ideally without the need to use lifts. After planning and marking the position of the BC-FMT and coil on the skin (Fig. 3a), a flap containing skeletal muscle and the periosteum for later coverage of the implant and a periosteal pocket for the implant were created. Then, the position of the BC-FMT was marked on the bone with the FP-sizer, a dummy supplied with the implant (Fig. 3b, c), followed by the drilling of a bone bed that should be at least 4.5 mm deep (Fig. 3d). The depth can be checked with the T-sizer, another dummy supplied with the implant (Fig. 3e). In children with low bone thickness, the dura was exposed but due to careful preoperative implant site selection there was no compression of the dura. Before fixation, the final position of the implant was checked with the FP-sizer, especially for verifying flat contact of the fixation wings to the bone, and the coil was bent in position (if needed, up to 90° in the horizontal and 30° in the vertical plane) and placed in the periostal pocket. Then, the BC-FMT was fixed with self-cutting screws followed by three-layered closure (Fig. 3f–h).

### Audiologic measurements

Audiological measurements included preoperative and postoperative air conduction (AC) and bone conduction (BC) thresholds. If AC and BC measurements could not be performed by the children, brainstem-evoked response audiometry (BERA) was measured instead. In addition, postoperative aided sound field audiograms (*S*_0_) and aided word recognition score in quiet (in % at 65 dB SPL presentation level) were determined. For assessing speech recognition, either the German Freiburg monosyllable test (20 monosyllables), the Goettingen child test for speech perception (10 monosyllables), or the Mainz speech test for children (10 monosyllables and disyllables) was used. The appropriate speech test was selected according to the childrens’ individual linguistic competence. All audiological tests were performed in a soundproof chamber with the speakers at 1 m distance to the subject. The contralateral ear was plugged and muffled if necessary. All postoperative tests were performed after the initial fitting of the processor which generally takes place four to five weeks after implantation.

### Statistics

Paired *t* tests were used to test for significant differences between preoperative and postoperative bone conduction thresholds. A regression analysis was performed to estimate the relationship between bone thickness and age at implantation. If not stated otherwise, mean ± standard deviation is shown. The pure tone average (PTA_4_) was calculated as mean values across the frequencies 0.5, 1, 2, and 4 kHz.

## Results

### Preoperative imaging

Preoperative imaging showed sufficient bone thickness (≥ 4.5 mm) for implantation of the second generation Bonebridge at the sinodural angle, the preferred location according to the manufacturer, in all patients’ ears. The Otoplan evaluations demonstrated that the areas with sufficient bone thickness for implantation of the second-generation Bonebridge are substantially increased in comparison to the first generation Bonebridge (Figs. 1, 2). Preoperative CT evaluations using Otoplan could potentially replace the manual assessment of bone thickness values, especially if the generated maps and resulting target location can be projected onto the actual patient during surgery (e.g., using navigation systems).

### Bone thickness

**Fig. 4:**
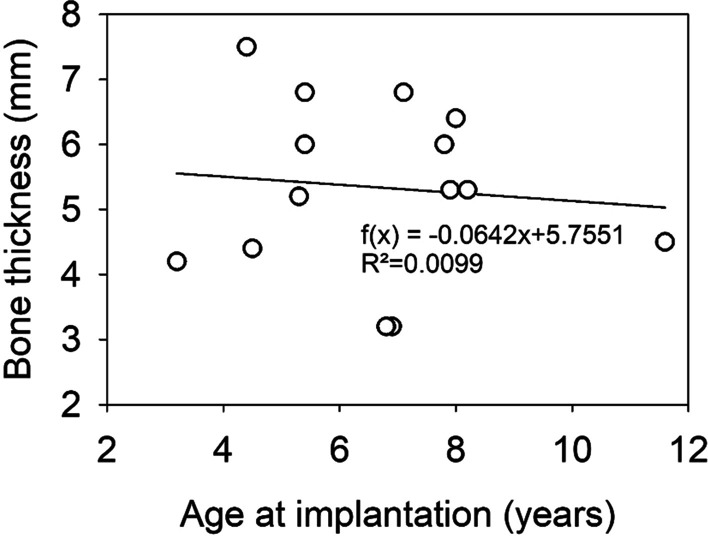
Individual minimal temporal bone thickness values (in mm) plotted over age at implantation (in years) for all 14 children implanted with the Bonebridge

Minimal thickness of the temporal bone of implanted ears ranged from 3.2 to 7.5 mm (5.3 ± 1.3 mm). No significant correlation was found between bone thickness (outcome variable) and age at implantation as predictor variable (Spearman rank order correlation, *p* > 0.05) for the selected children in the age group of 3 to 12 years. Although the slope of the regression line was found to be significant (slope *b* = − 0.0624, *p* = 0.0006), the amount of variance explained by the model (*R*^2^ = 0.0099) and the correlation coefficient (*R* = 0.099) are low (Fig. 4). In children, who were implanted bilaterally, the difference in bone thickness between the left and the right ear ranged between 0.2 and 3.6 mm (Table 2).

**Table 2:** Minimal bone thickness of left and right ear of bilaterally implanted children

Patient ID	Min. Bone thickness (mm)		Difference
	Right	Left	
P05	6.0	6.8	0.2
P06	6.8	7.5	0.7
P07	3.2	4.2	1
P09	6.8	3.2	3.6

### Surgical outcome

In all patients included in this study, the implant was placed at the sinodural angle as suggested by the manufacturer. The surgical procedure was fast and easily performed. The surgical time of a standard unilateral implantation procedure in patients with the BCI 602 (73.4 ± 6.9 min, *n* = 7) was reduced by 14.6 min compared to patients implanted with the BCI 601 (88.0 ± 23.1 min, *n* = 5), however results were not significant (*t* = 1.598, *p* < 0.141, Student’s *t* test). Furthermore, no lifts had to be applied in any of the children, not even in the three children under the age of five included in this study.

The surgical procedure was successful in all patients with no intraoperative complications. However, two postoperative complications were recorded. One postoperative complication, a swelling over the implant, was experienced by child P03 one week after the implantation. The swelling was not associated with either redness, pain, or fever, and resolved after treatment with a circular bandage and prophylactic antibiotic therapy. Another child (P08) was reimplanted after the explantation of the first BCI 602 before the initial fitting because of an occurring wound infection. During the revision surgery, the first implant was removed and the necrotic skin was replaced with a combined skin and temporalis muscle flap. The data for this retrospective study was obtained from the second Bonebridge BCI 602 implant which was implanted seven months after the revision surgery without any preoperative and postoperative complications. No complications occurred in BB implanted children under 5 years of age.

### Audiological results

**Fig. 5:**
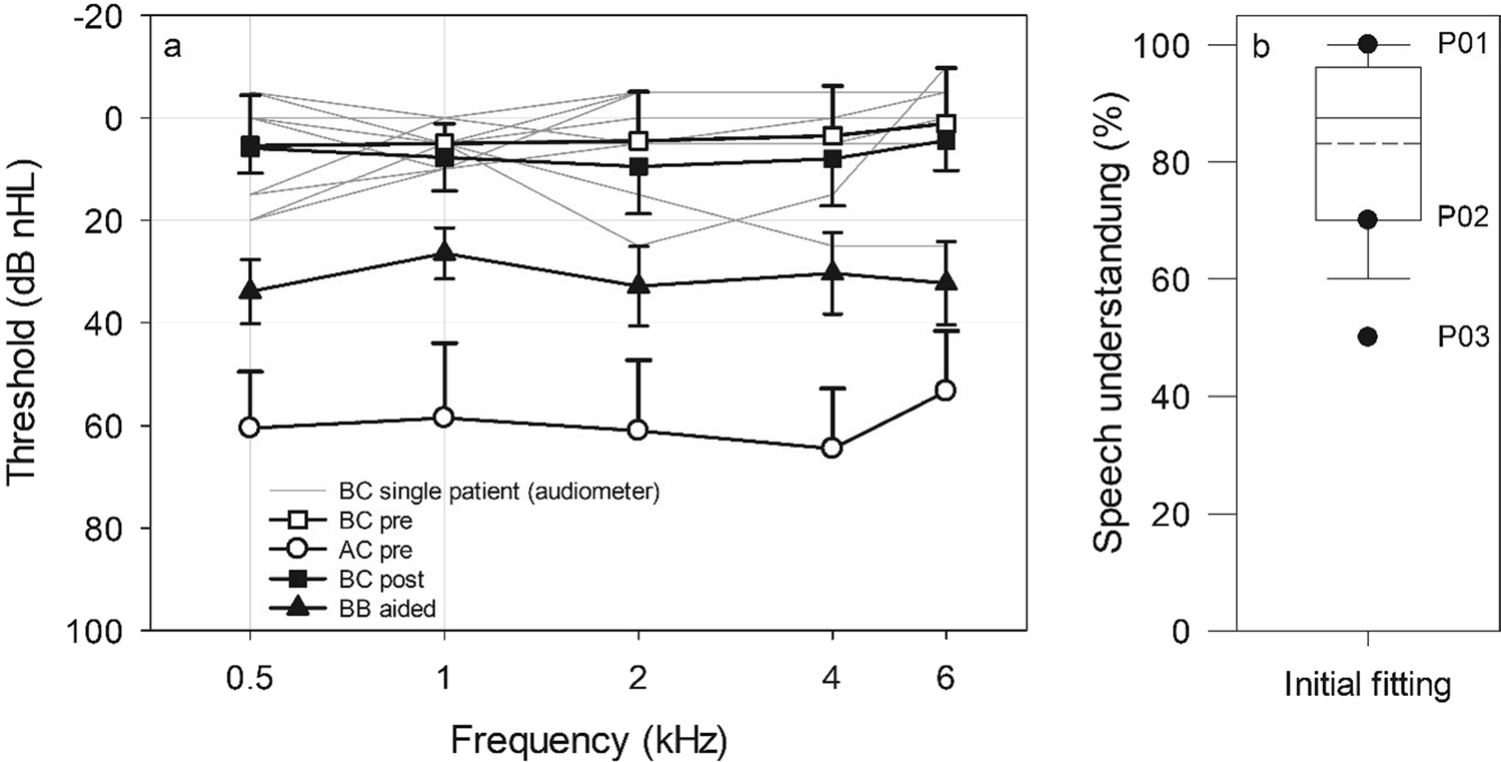
Displayed are **a** preoperative air conduction (AC) and bone conduction (BC) thresholds as mean value (± standard deviation) or single patient data, postoperative BC threshold, Bonebridge aided threshold (*n* = 10) and postoperative speech understanding with Bonebridge after initial fitting (*n* = 14) and **b** the mean (dashed line) and median (solid line) speech understanding of *n* = 14 children and single patient data of three children under the age of 5. Asterisks indicate significant differences between preoperative and postoperative BC thresholds (*p ≤ 0.05)

The average preoperative and postoperative BC thresholds (*n* = 10) were 4.3 ± 6.0 dB HL and 7.4 ± 5.9 dB HL, respectively (Fig. 5). Mean changes in BC thresholds across frequencies from preoperative to the time of initial activation ranged from 0.5 dB at 0.5 kHz to 5.0 dB at 2 kHz. Minor changes in postoperative BC threshold of ≤ 5.0 dB with a significant decline were observed at 2 kHz (+ 4.5 dB HL, *t* = − 2.535, *p* = 0.0319) and 4 kHz (+ 5.0 dB HL, *t* = − 2.586, *p* = 0.0294). After the initial fitting of the processor, 4.7 ± 0.7 weeks after the implantation in 13 children and after 11.1 weeks in one child, all children (n = 14) achieved an average Bonebridge-aided threshold of 30.9 ± 5.2 dB HL (ranging from 26.4 ± 5.0 dB HL at 1 kHz to 33.9 ± 6.3 dB HL at 0.5 kHz) and an average word recognition score of 83.2 ± 14.8% (median 87.5%) (Fig. 5). The three patients (P01, P02, and P03) younger than the age of 5 achieved a WRS of 100%, 70% and 50%, respectively (Fig. 5).

## Discussion

This study demonstrates that the second generation of the transcutaneous bone conduction implant Bonebridge can be implanted safely in children under the age of 12. Within this study, 14 children (18 ears) under the age of 12 received a unilateral or bilateral implantation of the second-generation Bonebridge. All implants could be placed in the sinodural angle without the necessity to use lifts. This beneficial surgical outcome was supported by preoperative imaging and the use of Otoplan to highlight all areas with sufficient bone thickness. In children with low bone thickness, the dura was exposed and in contact with the BC-FMT, but due to careful preoperative implant site selection, there was no compression of the dura.

The Otoplan images also nicely demonstrate the increased areas of sufficient bone thickness for the second-generation Bonebridge with the decreased thickness of its BC-FMT in comparison to the first generation. The downsized transducer allows for implantation of younger patients as demonstrated by the three implanted children under 5 years included in this study. Our preliminary results are supported by findings of a retrospective analysis of CT scans of mastoids from children and adolescents (Wenzel et al. 2020). They estimated that the BCI 602 can be implanted in 70% of the temporal bones of children between 3 and 5 years, whereas the BCI 601 implant could not be virtually implanted in children under 5 years of age. Another advantage of the BCI 602 is that the surgeon potentially does not need to use lifts at all. In all 14 children with Bonebridge BCI 602, no lifts were necessary due to reduced height of the transducer. In comparison, in 6 children, between 4.7 and 10.3 years at implantation (of which 3 children were bilaterally implanted) with 9 BCI 601 implants from our clinic, 1- or 2-mm BCI lifts had to be used in 5 out of 9 cases (55.6%) during implantation. Omitting lifts reduces the number of surgical steps and surgery time. The surgical procedure was successfully applied in all patients with no intraoperative complications. A minor postoperative complication, most probably a seroma, occurred in one child (7.1%) and did not require surgical therapy. One major postoperative complication (7.1%), a wound infection with skin necrosis that required revision surgery, highlights the necessity for a good wound management especially in younger children with thinner skin and temporalis muscle. No complications occurred in BB implanted children under 5 years of age. Our postoperative complication rate with the BCI 602 in children is low and similar to findings of other studies on children and adolescents implanted with the BCI 601. Bae et al., reported one major complication (16.7%) in six implanted children under 5 years [22], while Ngui and Tang, inform about one minor complication (16.7%) in six implanted children with congenital aural atresia between 5 and 18 years, a mild infection at surgical side treated with antibiotics resolved after 1 week [23]. One minor complication, a hematoma and pressure sensitivity behind the ear after saxophone practice, was found in three implanted children between 10 and 16 years [24]. In a larger study group (*n* = 32) including seven children below 16 years, four minor complications (12.5%) and one major complication (3.1%) were presented [25]. In a comparison study with *n* = 5 BCI 601 and *n* = 7 BCI 602 implanted children between 6 and 19 years, three postoperative not procedure-related complications [BCI 602] and one major revision (9.1%) [BCI 601] were described [26].

All children implanted with the Bonebridge achieved beneficial audiological outcomes with no deterioration of residual hearing. Minor changes in postoperative BC threshold occurred in the mid-frequencies. Although the decline in postoperative BC threshold was significant at 2 and 4 kHz, changes of ≤ 5.0 dB were within the accuracy limit of bone conduction measurements (± 5 dB) and considered clinically irrelevant. Furthermore, a median speech recognition score of 87.5% directly after initial fitting and individual scores between 70 and 100% (*n* = 13) and 50% in only one of the young patients is evidence for effective hearing rehabilitation with the Bonebridge implant. The mean aided threshold of 30.9 ± 5.2 dB HL is comparable to the findings of other studies reporting results with the BCI 601 in adults and children (28.2 ± 8.2 dB HL, *n* = 11 [27];) and with the BCI 602 in children (34.4 ± 8.9 dB HL, *n* = 22 [28]).

Within the observed age group of 3–12 years, bone thickness was not correlated with age, and the proportion of variance in the bone thickness explained by age was found to be very low. Our results hence show that age is not a reliable predictor for bone thickness. Furthermore, the bone thickness of the mastoid in a single subject can vary greatly between both sides, especially in cases of outer ear malformations. In four of the twelve children, which were implanted bilaterally, the bone thickness was determined for both ears. The difference between the left and the right ear varied greatly between children ranging from 0.2 to 3.6 mm. The child with the biggest difference in bone thickness suffered form Treacher–Collins Syndrome, a syndrome leading to complex malformations. Thus, eligible candidates for Bonebridge implantation should not be selected based on their age but on bone thickness individually determined for each ear.

## Conclusion

With adequate preoperative workup, this device can be safely implanted in children and even children under 5 years with a beneficial postoperative audiological outcome. We further suggest the use of bone thickness instead of age as the main indication criteria for Bonebridge implantation.

## Supplementary Information

Below is the link to the electronic supplementary material.

Supplementary file1 (PDF 71 KB)

## Data Availability

The data that support the findings of this study are available from the corresponding author upon reasonable request.
