# Supplementary material for: Surgical and audiological outcomes with a new transcutaneous bone conduction device with reduced transducer thickness in children
Source: Eur Arch Otorhinolaryngol. 2023 Mar 31;280(10):4381–9. doi: 10.1007/s00405-023-07927-9 (PMC10477095; doi:10.1007/s00405-023-07927-9)
Supplement: Supplementary file 1 — Supplementary file1 (PDF 71 KB) [file 405_2023_7927_MOESM1_ESM.pdf]

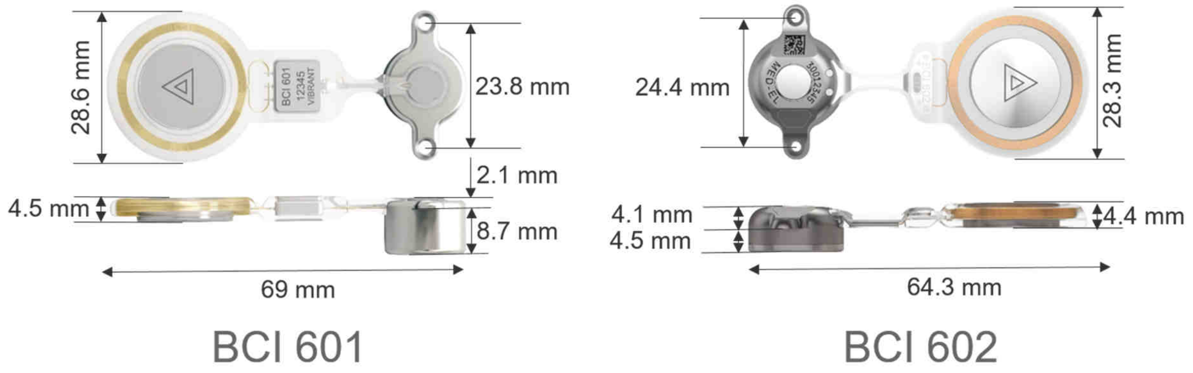

Online Resource 1
Comparison of the dimensions of the first (left) and second generation (right) of the implant (courtesy of MED-EL)
